# mHealth Self-Report Monitoring in Competitive Middle- and Long-Distance Runners: Qualitative Study of Long-Term Use Intentions Using the Technology Acceptance Model

**DOI:** 10.2196/10270

**Published:** 2018-08-13

**Authors:** Sara Rönnby, Oscar Lundberg, Kristina Fagher, Jenny Jacobsson, Bo Tillander, Håkan Gauffin, Per-Olof Hansson, Örjan Dahlström, Toomas Timpka

**Affiliations:** ^1^ Athletics Research Center Department of Medical and Health Sciences Linköping University Linköping Sweden; ^2^ Rehabilitation Medicine Research Group Department of Health Sciences Lund University Lund Sweden; ^3^ Department of Behavioural Sciences and Learning Linköping University Linköping Sweden

**Keywords:** running, mHealth, health technology, diagnostic self-evaluation, remote sensing technology, self-evaluation programs, qualitative research

## Abstract

**Background:**

International middle- and long-distance running competitions attract millions of spectators in association with city races, world championships, and Olympic Games. It is therefore a major concern that ill health and pain, as a result of sports overuse, lead to numerous hours of lost training and decreased performance in competitive runners. Despite its potential for sustenance of performance, approval of mHealth self-report monitoring (mHSM) in this group of athletes has not been investigated.

**Objective:**

The objective of our study was to explore individual and situational factors associated with the acceptance of long-term mHSM in competitive runners.

**Methods:**

The study used qualitative research methods with the Technology Acceptance Model as the theoretical foundation. The study population included 20 middle- and long-distance runners competing at national and international levels. Two mHSM apps asking for health and training data from track and marathon runners were created on a platform for web survey development (Briteback AB). Data collection for the technology acceptance analysis was performed via personal interviews before and after a 6-week monitoring period. Preuse interviews investigated experience and knowledge of mHealth monitoring and thoughts on benefits and possible side effects. The postuse interviews addressed usability and usefulness, attitudes toward nonfunctional issues, and intentions to adhere to long-term monitoring. In addition, the runners’ trustworthiness when providing mHSM data was discussed. The interview data were investigated using a deductive thematic analysis.

**Results:**

The mHSM apps were considered technically easy to use. Although the runners read the instructions and entered data effortlessly, some still perceived mHSM as problematic. Concerns were raised about the selection of items for monitoring (eg, recording training load as running distance or time) and about interpretation of concepts (eg, whether subjective well-being should encompass only the running context or daily living on the whole). Usefulness of specific mHSM apps was consequently not appraised on the same bases in different subcategories of runners. Regarding nonfunctional issues, the runners competing at the international level requested detailed control over who in their sports club and national federation should be allowed access to their data; the less competitive runners had no such issues. Notwithstanding, the runners were willing to adhere to long-term mHSM, provided the technology was adjusted to their personal routines and the output was perceived as contributing to running performance.

**Conclusions:**

Adoption of mHSM by competitive runners requires clear definitions of monitoring purpose and populations, repeated in practice tests of monitoring items and terminology, and meticulousness regarding data-sharing routines. Further naturalistic studies of mHSM use in routine sports practice settings are needed with nonfunctional ethical and legal issues included in the evaluation designs.

## Introduction

Middle- and long-distance running is one of the most popular forms of physical exercise worldwide, and running competitions attract millions of spectators in association with city races, world championships, and Olympic Games [1]. It is therefore a major concern that ill health and pain, as a result of sports overuse, are common causes of lost training and decreased performance among long-distance runners [2-4]. Recent research suggests that early detection of overuse syndromes may be achieved through observation of indicators at levels other than tissue damage. One way to identify early indications of decreased performance among runners is to continuously record external loads and then evaluate how the runners are affected by these loads [5]. However, such passive monitoring requires extensive technical resources, and it is also challenging to analyze data monitoring from different individuals in a meaningful way. As an alternative, self-report measures have been described as adequately reliable and sensitive compared with other ways of measuring athletes’ responses to training load [6]. Athlete self-reporting on training and health status, using the World Wide Web, is simple to administer and inexpensive [7,8]. Despite its potential, research on implementation of mHealth self-report monitoring (mHSM) among runners is sparse [9].

**Figure 1:**
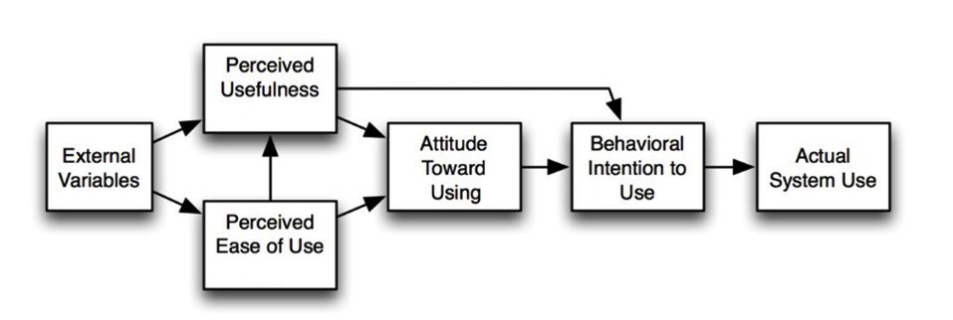
The original version of the Technology Acceptance Model used in the present study [12].

In any setting where novel technology is incorporated with established practices, knowledge of user acceptance is important [10]. The Technology Acceptance Model (TAM) was developed to help analyze, explain, and modify computer usage behaviors in a framework based on the Theory of Reasoned Action, as seen in Figure 1 [11,12]. In this model, it is assumed that a person’s willingness to use a technical system or device is mediated by attitudes toward system use that are founded on the system’s perceived usability and usefulness. Usability is defined as the level at which a person finds using the system to be free of effort, whereas usefulness is the extent to which the person finds that using the system enhances his or her performance of important tasks. The attitude concept in TAM involves a person’s beliefs about the consequences of adopting the novel technology and whether these consequences are regarded to be positive or negative. These beliefs influence the reasoning that determines the person’s inclination to use or not to use technology [11]. According to TAM, perceived ease of use, perceived usefulness, and attitudes toward the consequences of using the technology thus shape use intentions that guide use behaviors. TAM also recognizes that usability and usefulness perceptions are influenced by preexisting contextual factors. More recent versions of TAM explicitly include concepts denoting external barriers to technology use, such as costs and maintenance [13].

In theory, monitoring by self-report measures has the potential to provide useful information for competitive runners exposed to high training intensities and large training volumes, predisposing them to running-related health problems [14,15]. However, the usability and usefulness of mHealth monitoring systems, based on such measures, have not been assessed among middle- and long-distance runners. The aim of this study was to explore individual and situational factors associated with mHSM acceptance and long-term use in competitive runners.

## Methods

### Study Design and Setting

This study was based on a pre-post intervention design and qualitative research methods [16]. The setting was an initiative by the Swedish Athletics Federation to monitor the performance and health of runners competing at middle, marathon, and ultramarathon distances. Following a development and test period, the ambition is to introduce mHealth monitoring and feedback as a regular component of coaching and medical support. The purpose of mHSM in this research was defined as follows: “to collect longitudinal training and health data to be used for individual-level feedback among coach- and self-directed runners.” Before data collection, a web survey design tool was used to develop specific mHSM apps for longitudinal data collection. Semistructured interviews were used for collection of data before and after use of the prototype apps. The qualitative interview data were structured, interpreted, and categorized using a thematic analysis and have been reported according to the consolidated criteria for reporting qualitative research criteria for reporting qualitative research based on interview data [17].

### Ethical Considerations

In accordance with the Swedish legislation, this study was subject to review by research ethics committees [18]. The project was planned and conducted in accordance with the ethical principles of the Declaration of Helsinki. Before inclusion in the study, oral and written information about the purpose of the study was provided and each participant gave his or her written informed consent. Participation in the study was voluntary. All study data were handled without breaching the integrity of individual athletes.

### Study Population

The study population was defined to include adult runners (>18 years) competing at the national level, in middle or longer distances, in elite or veteran categories. Purposive sampling was used to ensure variation in gender, age, and running events. For recruitment of participants, 3 running clubs in Sweden were contacted through their head coaches. After discussions with their runners, all clubs accepted, at the group level, to participate in the study. The clubs offered runners support both by individual coach-directed schemes and through group-level coaching for self-directed runners. Individual invitations were then sent by email. According to the saturation principle, the recruitment of participants for individual interviews continued as long as new aspects appeared in the data.

### mHealth Self-Report Monitoring Software

**Figure 2:**
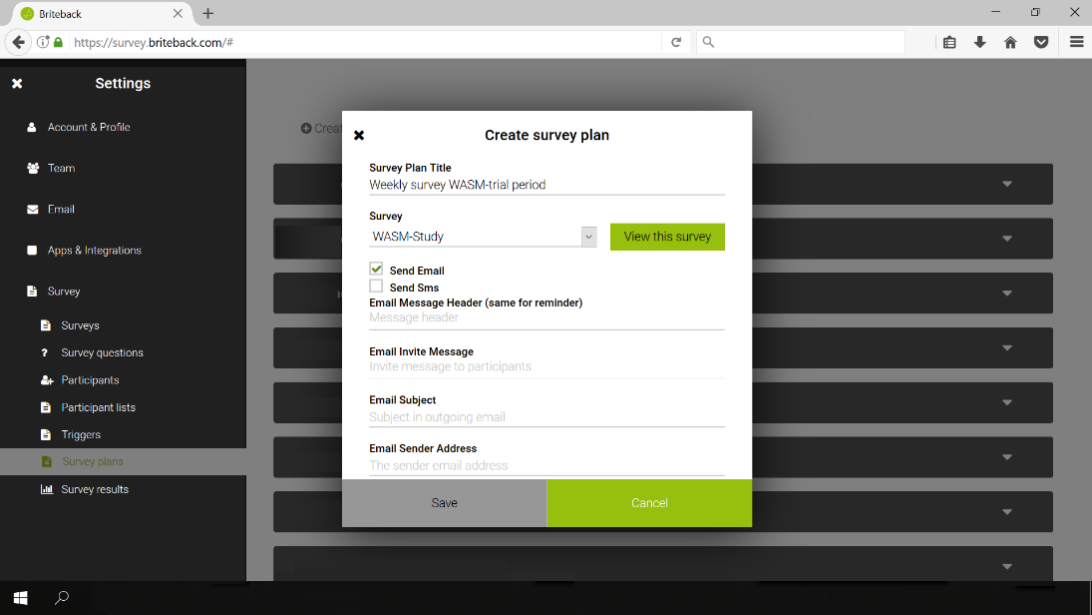
Display from the Web platform (Briteback) used to create and schedule weekly monitoring (Source: Sara Rönnby, Athletics Research Center, Linköping University).

The mHSM software used in this study was developed on a Web platform , Briteback AB, Norrköping, Sweden) where Web survey apps can be created and handled. For this study, 2 surveys asking for health and training data were developed and adapted for track and marathon runners. Both surveys included questions about the runners’ training and if they had experienced health issues. The surveys distributed to the marathon runners were slightly more detailed regarding training load, and the surveys provided to track runners were more detailed regarding health issues. After the survey items had been compiled for the monitoring events, the distribution of the events was sequenced in a monitoring plan, as seen in Figure 2, where the timing of repeated longitudinal monitoring was scheduled. The runners who did not complete the survey in 3 days received an automatic reminder by email with a new link to the survey.Automated system-generated statistics were provided for the researchers immediately after reporting of data.

**The 6-step method used for thematic analysis of the interview data [ 19].**

Thematic analysis

1. Familiarization with data
2. Initial code generation
3. Searching for themes
4. Reviewing themes
5. Naming themes and categorizing them according to Technology Acceptance Model
6. Producing report

### Data Collection

The first set of semistructured interviews were conducted by two authors (OL and SR) before mHSM was initiated. An interview guide with open-ended questions that covered the main aspects of TAM was used. The interviews lasted for about 30 minutes and were audio recorded. The preuse interviews investigated the experience and knowledge of mHealth monitoring, followed by the runners’ thoughts on possible benefits and side effects. The postuse interviews investigated perceived usability and usefulness, attitudes toward nonfunctional issues, and intentions to adhere to longitudinal monitoring. In addition, the runners’ trustworthiness when using the system was questioned, and thoughts on improvements and what they would want to change to optimize the system for their training were investigated. After the interviews, the pre- and posttrial interviews were transcribed verbatim.

### Data Analysis

A deductive thematic analysis [19] was performed using TAM as the theoretical foundation, as seen in Textbox 1.

To familiarize themselves with the data, the transcribed interviews were read through repeatedly by three authors (SR, OL, and TT). With the study aims in mind, the most relevant parts of the data were identified, and key parts were extracted from the individual responses, from which meaning units were identified regarding their content and context. The meaning units were interpreted at a semantic level rather than a latent level. Codes were produced consisting of keywords that captured the essence of the meaning units. The codes were used to gain understanding and to compare meaning units. The coded meaning units were then grouped into categories. After reviewing the categories and adjusting some of them, the categories were grouped into themes named using a short sentence. These themes were finally contextualized and classified according to TAM (Textbox 1) in a process that included all authors.

## Results

Data were collected from 20 runners (9 males and 11 females) aged between 20 and 64 years (Table 1). Most runners reported previous monitoring experience, mainly the use of training diaries for communication with their coach. Also, some self-directed runners without a personal coach stated that they had practiced data collection during their training. More often than not, the coach-directed runners planned their training down to exact exercises, whereas the self-directed runners scheduled their training in a less detailed way (eg, a rough weekly plan of the amount of training).

The runners’ previous mHSM experience was scarce. None of them had used more complex documentation and analysis tools than an mHealth training diary. A male runner’s description of his experiences was typical for the runners.

I have looked at some online tools, I may well say. Online coaches, those kind of automated, for instance. But it's nothing that I have followed regularly. It is mostly really training diaries that I have used.

During the mHSM trial period, 19 participants provided monitoring data every week; one runner failed to provide data for the final week because of technical issues (Table 2). Most participants responded in the first couple of days for most of the weeks in the trial period. However, 15 of 20 runners received a first reminder (sent out 3 days after the initial weekly survey).

### Predispositions and External Factors

To be motivated for participation in mHSM, the runners explained that they needed to expect a positive balance between immediate burden and future reward. A long-distance runner explained:

It depends on how much data I should report, that is, how many questions I have to answer. The more questions, the less keen I am to report.

Regarding rewards, the runners envisioned new services that could be provided through mHSM, such as ubiquitous availability of support to interpret trends and long-term planning of running schedules. Integrated graphical displays of training patterns and competition performances were asked for by a middle-distance runner:

I would like to more easily be able to see trends and patterns over time. Such as ‘Oh, here I trained so and so, and then I got these results.’ It would provide a better overview of how I've trained and what it provides in terms of consequences, both regarding results but also injuries and other things.

**Table 1:** Characteristics of participating runners.

Participants	Sex	Age (years)	Event(s)	Coach directed	Detailed schedule	Monitoring experience	mHealth self-report monitoring experience
1	Male	27	10-21 km	Yes	Yes	Yes	No
2	Female	20	5-21 km	Yes	Yes	No	No
3	Male	24	1500 m to 5 km	Yes	Yes	Yes	No
4	Male	24	5-21 km	No	No	Yes	No
5	Male	21	5 km	No	Yes	Yes	Yes
6	Female	25	Middle distance	Yes	Yes	No	No
7	Male	27	3-5 km, cross country	No	No	Yes	Yes
8	Male	24	10-21 km	No	No	No	No
9	Female	34	5-10 km	Yes	Yes	Yes	No
10	Male	24	10-42 km	No	No	No	Yes
11	Female	55	Marathon	No	No	No	No
12	Male	57	Marathon	No	No	Yes	No
13	Female	56	Marathon	No	Yes	No	No
14	Female	64	Ultramarathon	No	Yes	Yes	No
15	Male	40	Marathon	No	Yes	Yes	No
16	Female	45	Ultramarathon	No	No	Yes	No
17	Female	55	Ultramarathon	No	No	Yes	No
18	Female	48	Ultramarathon	No	Yes	Yes	No
19	Male	46	Marathon	No	No	Yes	No
20	Male	31	Marathon	No	No	Yes	No

Nonetheless, in parallel to the visions of new services and uses, concerns were also expressed that the playful aspect of participating in the sport might disappear. One runner clarified:

Somehow, the more you record about your training and the keener you are on structure and control, the greater is the risk that the spontaneity and joy will diminish or disappear. I'm running and working out mainly because I think it is so nice and fun. Yes, in some way you can feel a little limited or controlled [by mHSM], and, if you take this too far, there is a risk that the feeling of freedom and joy of running disappears in part I think.

A related concern was that mHSM could introduce unnecessary stress. In particular self-directed runners, who less often discuss their training and competition schedules with a coach, were seen to be at risk. A self-directed long-distance runner explained:

Some people may become slaves under the system, and get anxious. ‘I was to have a 150 km week’ and then you find that you just reached 139 km and then you feel bad because of that.

Other runners envisioned that mHSM, in the future, would allow large quantities of data to be collected from many runners. Access to these large datasets would be interesting also for parties outside the traditional athlete-coach setting. The runners were clear that the new external uses of their data should not be allowed without their permission, highlighting the “market value” of the data collected:

Well, if [the data] can be linked to me as a person, I think no one should have access to [my data] without my knowledge, but it may be okay with my consent. I would lett physicians and coaches and others use these data. However, not just anyone, it should be based on my consent.

### Perceived Ease of Use

The mHSM apps were considered easy to use and understand, and most runners did not experience technical difficulties during the test period. They stated that use was easier and less demanding than expected. One of the middle-distance runners expressed a typical opinion:

The survey itself was not as long as I had feared it would be. It went a lot faster than I expected, but that's of course linked to my expectations. I experienced it as quick and easy to fill in.

However, although the monitoring items were understandable, they still could be perceived as problematic. For example, one runner had difficulties differentiating between well-being with regard to running achievements and well-being in general:

It is hard to know how to report some features. They are kind of subjective, for example, ‘How you do feel today?’

**Table 2:** Overview of runners’ monitoring behaviors.

Participants	Monitoring compliance	Response lag (days)	Reminders issued	Device used	Human-computer interface concerns^a^
1	Yes	1-3	1	Mobile phone	No
2	Yes	1-2	1	Mobile phone	No
3	No^b^	1-3	2	Computer	Yes
4	Yes	1-2	0	Computer	No
5	Yes	1-2	0	Mobile phone	No
6	Yes	1-2	1	Computer	No
7	Yes	1-2	1	Mobile phone or tablet	Yes
8	Yes	1-4	3	Mobile phone	No
9	Yes	1-3	1	Computer	No
10	Yes	1-2	1	Computer	No
11	Yes	1-5	2	Computer	No
12	Yes	1-2	0	Computer	Yes
13	Yes	1-3	1	Computer	No
14	Yes	1-3	1	Mobile phone	Yes
15	Yes	1-2	0	Mobile phone	No
16	Yes	1-3	1	Mobile phone or tablet	No
17	Yes	1-2	0	Computer	No
18	Yes	1-2	0	Mobile phone or tablet	No
19	Yes	1-3	1	Mobile phone	No
20	Yes	1-2	0	Computer	No

^a^Problems with the human-computer interface for data entry.

^b^Technical problems in the final week.

The timing of survey distribution was found to be important when responding to the weekly questions in the midst of everyday chores. The runners tried to find a situation where they could routinely respond to the survey without being disturbed:

I replied probably almostright away. It was sent out just before Iwere going to sleepso then I did it before I went to bed.

Despite the mHSM apps being perceived as easy to use, responding could still be complicated by the fact that safe access to the internet was unnecessarily difficult. One of the runners explained:

Web developers [sigh]… They are going to complicate everything. The purely IT-related parts have to be both safe and easy. Best would be if you could log in via a social network or email, so you do not have 3 billion passwords everywhere.

### Perceived Usefulness

The runners expressed that mHSM was useful in general but emphasized that the items being monitored had to be formulated so that they were relevant to improving their running. A certain level of detail in the training data was needed to offer this usefulness, although the effort had to stay as low as possible. One middle-distance runner’s view summarized the general perception of the monitoring scope:

The length of the survey was really good. Comprehensive, but not too extensive. It cannot be too long if you're going to fill it in frequently...Multiple choice questions, ratings, and such are good. It becomes fairly accurate and time efficient.

Opinions varied on the useful levels of sports load recording, but the runners agreed that the balance between information detail and the recording burden was important. Another runner explained:

I would probably want it more detailed; the survey was a little too brief for it to be really useful. But more detail would be more demanding. However, as long as you can feel motivation and purpose, there is no problem with more extent.

However, in some circumstances, there were differing opinions about what data to report (eg, recording training volume as running distance or time) and difficulties with the interpretation of concepts (eg, whether subjective well-being should encompass daily living as a whole or only the running context). The runners also pointed out questions and functions that were not included in the monitoring. For example, one runner asked for more open reporting formats:

The survey questions were very specific. Yes, running is what it was about, so that was no problem [to report]. But then using skiing and swimming as alternative forms of exercise [were also asked for]. There may well be many other relevant alternative forms of exercise that runners use.

### Attitudes Toward Using

Based on that, the runners found mHSM easy to use and potentially useful, their reasoning about adoption consequences came to focus on concerns about nonfunctional issues, mainly privacy and integrity. Regarding access to monitoring data, individuals directly supporting the runners and researchers were generally tolerated. However, the attitudes associated with access privileges differed between training and health data; data on personal health and mental well-being were considered more sensitive. One runner explained:

I thought about the questions regarding personal health, which are quite private. I had that in mind when I answered the surveys. A running diary can be really personal. Then I found that integrity is very important. Everybody may not want to show how they train or how they feel.

However, the perceptions of integrity breaches were contextual and relatively specific. A middle-distance runner clarified:

It could have been [sensitive for runners] if anything about mental health aspects was included, but it was not. There were questions only about general well-being and nothing about ‘Have you visited a psychologist?’ No, it would only be [sensitive] if deeper issues such as performance anxiety and depression were asked about. But such questions were not included.

A few of the runners suggested that if the information was made anonymous, access could be completely open. Regarding training data access, there were notable differences between different groups of runners. The typical standpoint among the runners was that training data could be shared relatively freely. A veteran marathon runner summarized her views after having used the mHSM system.

I feel that it does not matter if anyone knows that I've gone to the gym 2 days a week for 5 weeks, which I actually did, and ran 20 km one week and 100 km next week. I do not think that would be an invasion of privacy for me.

The deviating opinions regarding data sharing and integrity were related to team selections. The most competitive runners requested detailed control over who in the sports organizations they belonged to, from clubs to federations, should be allowed access to their data. One of these runners stated:

No, I believe that selections and so on should be based on performance and other ways [than monitoring]. It is difficult, I think, to associate competition results with this type of statistics and data. No, no I do not think it would be reasonable.

A typical argument for objecting to sharing data with team managers was that the runner was concerned that it would lead to untruthful self-reporting, that is, competitors for team selections would overstate training and downplay injuries.

I would not like it to influence selection to competitions and national teams. There should not be any reason not to be honest in your training diary or such. But all in your personal team should have access, if relevant. Otherwise I prefer it not spread.

Nonetheless, the runners also stated that they were truthful when providing data, as is shown in the following examples:

I tried to be very honest, there’s no reason to lie, it’s not a competition that way.

Since I keep a paper backup [for myself] as well, I know that [the data I provide] are honest [and accurate].

### Behavioral Intention to Use

The runners were willing to use mHSM for extended periods of time, provided that the monitoring was adjusted to their personal settings and feedback needs. Reporting on a weekly basis was preferred. One runner explained:

At least once a week. Otherwise, you start to forget. Above all, you forget how it felt. It would be most favorable to report each session but then it can be difficult to get it done.

mHSM technology in the runners was regarded as potentially beneficial for their personal development as athletes, for example, for performance improvement through injury prevention. Even so, the runners highlighted the balance between cost and benefit when considering use over extended periods of time:

If you think it is meaningful, it is time well invested considering what you can get out of it, as long as the system is easy to use.

The perfect balance between usefulness and burden was not the same for different categories of runners. However, overall, the runners found that acceptable reporting habits could be achieved.

## Discussion

### Principal Findings

In this qualitative study based on TAM, we found that variations in intentions to adopt mHSM among competitive middle- and long-distance runners could be explained by the perceived usefulness of the technology rather than its usability. The overall system design and the monitoring content were regarded as more important for adoption than specific utilities of the human-computer interface. Moreover, contextual nonfunctional issues, such as control of access to the collected data, influenced use intentions among the most competitive runners. These finding imply that acceptance of mHSM in routine settings in competitive runners will require clear definitions of purpose and user populations, meticulousness regarding data-sharing routines, and formative evaluations of the monitoring content in each specific app.

### Usefulness of mHealth Self-Report Monitoring

We found that the competitive runners needed to see a positive balance between immediate burden and future reward to be motivated for mHSM. Among self-directed runners, the burden of analyzing the data and using the results for their own health maintenance and performance improvement may be overwhelming. However, these athletes also need to gain a positive balance from a monitoring process to be sufficiently motivated to record data [20]. Consequently, being able to effortlessly generate interesting output from mHSM data analyses, such as graphical displays of performance and health trends, is particularly important for self-directed athletes. In comparison, for coach-supported runners, the burden of supplying mHSM data is more likely to be balanced by factors such as improved coordination of training management [21]. The perceptions of the members of the support team, in particular, the coach, are therefore important to consider when assessing the usefulness of mHSM for this category of runners.

The accuracy of the self-reported data is a related concern. Although the runners generally stated that they supplied accurate data, some athletes indicated that they occasionally guessed or made estimations. Acquiescent responding or indiscriminate agreement irrespective of survey content [7,22] may thus affect the quality of mHSM data among runners. In addition, conscious bias may occur. For instance, coach-supported athletes may report favorable data and underreport unfavorable data to gain selection, that is, “faking good” [23]. However, the validity of objective recording of physical loads, such as accelerometer data, has recently been questioned [24], and self-reporting has been shown to be the favored monitoring method regarding well-being and health influencing athletes’ performance [21,25]. This study did not analyze the validity of the reported training load data or that of the well-being and health data. The quality of self-reported data for these parameters should be further assessed among both self- and coach-directed athletes.

### Nonfunctional Issues

The finding that the most competitive runners were more concerned about access to their data can be compared with that of a recent study [8] that reported that coach-supported athletes were less concerned than self-directed athletes about data being secure and not misused. This lack of concern among coach-supported athletes was interpreted to reflect either a lower subjective importance of data sharing compared with the other factors or a particularly positive social environment in the study setting. However, other studies involving coach-supported athletes have reported concerns about athletes reporting their injury data to coaches [23]. In individual sports such as middle- and long-distance running, athlete selection for major competitions and teams usually takes place above the personal coaching level. The critical circumstance influencing attitudes toward data sharing in this setting thus appears to be athlete ranking and selection and not the coaching relationship per se. From these observations, we infer that ethical issues associated with mHSM in competitive runners cannot be evaluated without first defining the exact purpose of the monitoring and describing the individuals and groups that will have access to the data. Therefore, we suggest that future studies of mHSM usefulness are performed in routine sports practice settings. This implies that nonfunctional ethical and legal issues also need to be included in evaluations and that their solutions are allowed to influence the results.

### Usability Issues

The finding that mHSM usability was associated with the structure of the survey items can be compared with experiences from electronic data collection on preparticipation health in association with athletics championships [26] and mHSM among Paralympic athletes [27]. In both these contexts, the athletes encountered few usability problems but expressed concerns about medical terminology and formulation of the survey items. In sports settings, where standardized questionnaires have been used for data collection from athletes, differences have been observed regarding the ability to interpret concepts and respond as intended [28]. We infer that adequate adaption of monitoring items with regard to the characteristics and heterogeneity of the monitored population is key to be able to attain meaningful and useful data from long-term mHSM. However, before addressing the items, decisions need to be made on what proportions of standardized questionnaires, specific variables of interest, and pragmatic measures should be included in the monitoring. Therefore, we recommend that when developing an mHSM tool for long-term use, sufficient time and effort is allocated to define the specific purposes and goals of the data collection, the design of the tool is adjusted to whether it is to be used by self-directed or coach-directed runners, and the individuals and groups that will have access to the data are carefully considered. Thereafter, instruments need to be chosen or customized monitoring items need to be formulated such that the intended users understand and are motivated to use them. The runners in this study were not directly (hands on) involved in the creation of the mHSM items. We agree with the recommendations from a recent Dutch study that providing athletes with a tangible take away benefit from mHSM is essential [29] but add that inclusion of runners early in the design process of the app is strongly desirable. Before wide dissemination of an mHSM app for long-term use, several test periods, when intended users try out both the technical system and the monitoring items, should be completed. At the end of each period, experiences should be collected and the app design updated. Availability of a flexible software environment, where survey items can be changed easily during pilot trial processes, is a necessity.

### Future mHealth Self-Report Monitoring Apps

Preintervention, the participating runners reported having routinely recorded and analyzed training load data but had almost no mHSM experience. Accordingly, several novel uses of mHSM technology were suggested after the trial period. Alternative ways of using mHSM than those addressed in this study have been reported in the scientific literature, for instance, rather than using mHSM data for self-direction or traditional coaching support, data analysis can be performed cooperatively in peer-to-peer learning processes. For professional runners, their sport is their main source of income. By including mHSM data in structured peer-to-peer communication, self-employed runners can share information about training conducted as well as planned training programs, competition calendars, and competition venues. Groups of runners can thereby establish a contextualized learning process based on cooperative discovery. Web technology and devices are then used as facilitators and mHSM data as references for learning by comparison. Evaluations have highlighted that professional runners find discussions and sharing of experiences with peers in chat forums stimulating [30]. Self-esteem and self-reliance were found to improve as a consequence of receiving feedback, analyzing that feedback, and using the results to make adjustments and increase performance. Peer-to-peer communication supported by mHSM can thus predispose athletes toward a learning process where runners access education at low cost and are simultaneously empowered through integration into a wider community of sporting peers [31]. Sharing of training and health data thereby becomes the basis for a highly contextualized performance enhancement program.

### Strengths and Limitations

This study has strengths and weaknesses that need to be taken into consideration when interpreting the results. A strength is that the study used a pre-post design, which allowed analysis of attitude stability over time. An important limitation is that the mHSM system used in the trial only covered the initial mHSM phases (to record and review data) [32]. Inclusion of the remaining aspects (to contextualize and act) would have required involvement of coaches and other staff supporting the runners. Such an extended evaluation should be performed using more recent versions of TAM that include aspects such as costs, maintenance, integrity, and privacy [33,34]. Another weakness is that in this study, as in many other qualitative studies, the study group was relatively small (N=20). Nonetheless, recruitment of participants continued until saturation of the data was reached. Also, every runner category has unique features and needs. Therefore, extrapolating experiences of using mHSM from one category of runners to other running contexts should be done with caution. This study addressed usability and issues that might occur when attempting to create an mHSM app for the long-term surveillance of competitive middle- and long-distance runners, and the results do not provide complete information for the design of such a system for other runner categories. However, this study still highlights important aspects that should be considered when designing mHSM tools in other areas of sports epidemiology.

### Conclusions

Adoption of long-term mHSM by competitive runners requires clear definitions of purpose and populations, extensive in practice tests of survey items and terminology, and meticulousness regarding data-sharing routines. We suggest that further naturalistic studies of mHSM should be performed in routine sports practice settings. This implies that nonfunctional ethical and legal issues need to be included in the evaluation designs and that the solutions to these challenges are allowed to influence the results.

## Abbreviations

mHSM: mHealth self-report monitoring
TAM: Technology Acceptance Model
